# Transfer learning with false negative control improves polygenic risk prediction

**DOI:** 10.1371/journal.pgen.1010597

**Published:** 2023-11-27

**Authors:** Xinge Jessie Jeng, Yifei Hu, Vaishnavi Venkat, Tzu-Pin Lu, Jung-Ying Tzeng

**Affiliations:** 1 Department of Statistics, North Carolina State University, Raleigh, North Carolina, United States of America; 2 Bioinformatics Research Center, North Carolina State University, Raleigh, North Carolina, United States of America; 3 Institute of Health Data Analytics and Statistics, National Taiwan University, Taipei, Taiwan; 4 Department of Public Health, National Taiwan University, Taipei, Taiwan; Emory University, UNITED STATES

## Abstract

Polygenic risk score (PRS) is a quantity that aggregates the effects of variants across the genome and estimates an individual’s genetic predisposition for a given trait. PRS analysis typically contains two input data sets: base data for effect size estimation and target data for individual-level prediction. Given the availability of large-scale base data, it becomes more common that the ancestral background of base and target data do not perfectly match. In this paper, we treat the GWAS summary information obtained in the base data as knowledge learned from a pre-trained model, and adopt a transfer learning framework to effectively leverage the knowledge learned from the base data that may or may not have similar ancestral background as the target samples to build prediction models for target individuals. Our proposed transfer learning framework consists of two main steps: (1) conducting false negative control (FNC) marginal screening to extract useful knowledge from the base data; and (2) performing joint model training to integrate the knowledge extracted from base data with the target training data for accurate trans-data prediction. This new approach can significantly enhance the computational and statistical efficiency of joint-model training, alleviate over-fitting, and facilitate more accurate trans-data prediction when heterogeneity level between target and base data sets is small or high.

## Author summary

Polygenic risk score (PRS) can quantify the genetic predisposition for a trait. PRS construction typically contains two input datasets: base data for variant-effect estimation and target data for individual-level prediction. Given the availability of large-scale base data, it becomes common that the ancestral background of base and target data do not perfectly match. In this paper, we introduce a PRS method under a transfer learning framework to effectively leverage the knowledge learned from the base data that may or may not have similar background as the target samples to build prediction models for target individuals. Our method first utilizes a unique false-negative control strategy to extract useful information from base data while ensuring to retain a high proportion of true signals; it then applies the extracted information to re-train PRS models in a statistically and computationally efficient fashion. We use numerical studies based on simulated and real data to show that the proposed method can increase the accuracy and robustness of polygenic prediction across different ranges of heterogeneities between base and target data and sample sizes, reduce computational cost in model re-training, and result in more parsimonious models that can facilitate PRS interpretation and/or exploration of complex, non-additive PRS models.

## Introduction

Polygenic risk score (PRS), first proposed in [1], is a quantity that aggregates the effects of variants across the genome and estimates an individual’s genetic predisposition for a given trait. PRS can be used to explore the polygenetic architecture of a trait, to predict disease risk, to identify groups of individuals with substantially increased risks, and to study shared polygenic signals between different traits.

PRS is typically calculated as a weighted sum of trait-associated alleles, where the weights are the estimated effect sizes of the alleles on the trait. PRS analysis usually contains two input data sets: (i) base data, containing summary statistics of association tests from GWAS such as the estimated regression coefficients with the traits and their corresponding p-values, and (ii) target data, consisting of individual-level genome-wide genotypes data and traits used for prediction. Accumulating evidence suggests that for a variety of complex traits, variants selected based on a large ancestry-diversified base data are expected to harbor high fractions of causal variants carried by the target data, yet their effect sizes may not be the same across base and target data sets [2–4].

Because not all genetic variants influence the trait and because SNPs are correlated due to linkage disequilibrium (LD), two types of selection approaches have been adopted to construct PRS using GWAS summary statistics from the base data. The first type constructs PRS purely based on GWAS summary statistics from marginal model, i.e., removing correlated redundant SNPs via linkage disequilibrium (LD) pruning/clumping and computing the weighted allele sums only on those SNPs whose GWAS p-values are lower than a certain threshold (e.g., pruning/clumping + thresholding (C+T) [5], PRSice [6], and PRSice2 [7]). In these approaches, *p*-value threshold is usually set as a tuning parameter and optimized by data. The second type considers a joint additive model of all SNPs to estimate the effect sizes and to account for SNP LD, either by imposing Lasso regularization (e.g., lassosum [8]) or prior distributions (e.g., LDpred [9], PRS-CS [10] and LDpred2 [11]). PRS based on joint modeling tend to have higher prediction accuracy [12] due to the increased accuracy in estimated SNP effects.

Given the utility of large-scale base data, it becomes more and more common to encounter the scenarios where the ancestral background of the base sample and target sample may or may not be perfectly matched. For example, in the CoLaus/PsyCoLaus study [13, 14], the target sample is composed of Switzerland Caucasians while the base sample is composed of general European descents from US, central Europe, southern Europe and northern Europe. In multi-ethnic PRS prediction, the base sample tend to comprise European descents in large sample size as well as descents from other populations including the target population in moderate sample size. Consequently, although causal variants in the target data are expected to be included in a larger set of causal variants carried by the base data, the same variant may have different effect sizes in the base and target data sets.

To accommodate the potential ancestral heterogeneities between base sample and target sample, in this work, we treat the GWAS summary information obtained in the base data as knowledge learned from a pre-trained model, and adopt a transfer learning framework to leverage the knowledge learned from the relatively ancestry-diversified base data to build the PRS of target individuals. Our proposed trans-learning framework consists of two main steps: (1) conducting false-negative control (FNC) screening to extract useful knowledge from the base data; (2) conducting joint-model training to integrate the extracted knowledge with the target training data for accurate trans-data prediction. In (1), as SNP effects may differ across base and target datasets, marginal screening for promising SNPs should not just focus on strong base signals, but should have good capacity for weak base signals as well. Therefore, we use FNC to ensure the retention of a high proportion of strong and weak base signals and, at the same time, effectively exclude noise variants that are distinguishable from base signals. This is crucial for the second step of joint-model training. To ensure computational efficiency and numerical stability, it is essential to only include a reduced set of SNPs when training the joint PRS model; however, if the majority of signals were not included in this reduced set, the prediction accuracy of the PRS model would be compromised. In (2), we use joint regression to train the target PRS model with individual-level target data on the reduced promising-SNP set, so as to ensure accurate SNP effect estimates that better reflect the underlying causal SNP architecture of the target sample.

We use simulation to illustrate the performance of the proposed transfer learning procedure. In settings with various overlapping proportions of signals between base and target data sets, the new procedure leads to a better PRS model in terms of achieving a higher predictive *R*^2^ with a more parsimonious model. Application to the CoLaus/PsyCoLaus GWAS of lipid plasma indicates that besides being substantially faster than C+T, lassosum, and LDped methods, the new procedure can achieve the highest predictive *R*^2^ while retaining the least number of SNPs in the PRS model. A parsimonious PRS model that includes fewer SNPs whilst maintaining the same or higher predictive *R*^2^ can facilitate PRS interpretation and enable downstream analyses with more complex modeling techniques, such as incorporating SNP-SNP interactions in polygenic prediction models as discussed in [15, 16]. We also explore interactive models with the lipid prediction in the CoLaus/PsyCoLaus study.

## Materials and methods

### Formulation of trans-data signals

Let S be the set of causal variants carried by the target data and $S^{+}$ be the set of signal variants carried by the base data. Signal variants in $S^{+}$ are defined as variants whose true effect sizes in the GWAS marginal model are not zero. In this paper, we use “causal variants” to refer to variants truly affecting the phenotype, and use “signal variants” to refer to those variants that have non-zero true effect sizes in a certain statistical model. While causal variants are considered signal variants, the reverse is not always true. For example, a non-causal SNP that is in high LD with some causal variants would be a signal variant in the GWAS marginal model, but would not be a signal variant in a joint model that accounts for the causal variants. We consider heterogeneities between base data and target data from two sources: (a) the nested ethnicity relationship between base and target samples, and (b) the possible distortion of signal variants information from the pre-trained GWAS marginal model. A general assumption on the trans-data information sharing can be

$$S\subseteq S^{+}.$$

That is, the causal variants in the target data are included in the set of signal variants in the more ancestry-diverse base data. $S^{+}$ could also include non-causal variants, which have non-zero marginal effects because of their dependence with the causal variants in LD.

The nested assumption of $S\subseteq S^{+}$ could be satisfied by using multi-ancestry base data, which can be obtained by conducting meta-analyses on multiple GWAS of different ancestral groups, such as the multi-ancestry GWAS base data considered in [17]. The multi-ancestry base samples may be predominantly from a specific ancestral group; hence it is possible that the effect of a causal variant in S gets diluted in the ancestrally diverse base data and becomes fairly weak in $S^{+}$. In addition, the effect sizes of a variant can differ between base and target data due to the use of marginal association models in base data. An ideal PRS method should address these issues, incorporate both strong and weak base signals and re-train PRS model with target data.

### Trans-data polygenic risk prediction

We propose a transfer learning polygenic prediction procedure which performs FNC marginal screening to effectively retain both strong and weak signals in $S^{+}$ and applies the learned information from base data to identify causal variants in S and improve PRS for target individuals. Different from the classical power analysis in hypothesis testing, FNC screening does not hinge on a pre-fixed control level of type I error (or some form of cumulative type I errors in multiple testing). Instead, FNC screening adapts to a user-specified level of false negative proportion (FNP) to facilitate more powerful discovery of weak signals while keeping out noise variants (i.e., non-signal variants) that are distinguishable from weak signals.

**Fig 1:**
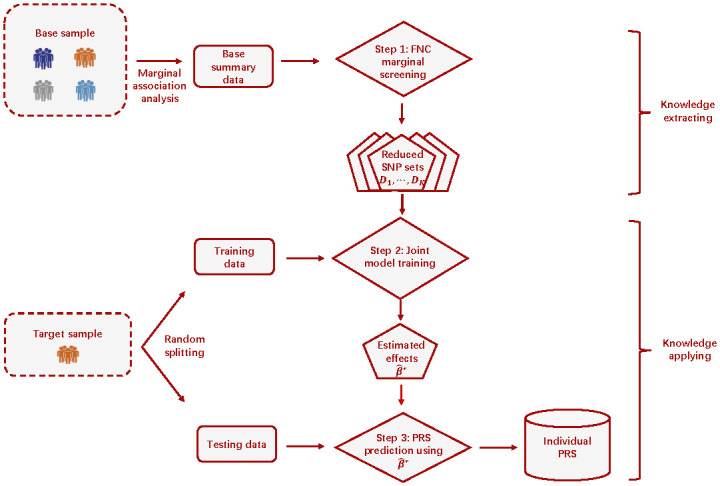
Overview of the PRS transfer learning procedure. Step 1: Given a pre-specified grid of control level for false negative proportion, {*ϵ*_1_, ⋯, *ϵ*_*K*_} (e.g., {0.02, 0.04, ⋯, 0.4}), apply false negative control (FNC) marginal screening to the base summary statistics and obtain *K* candidates of reduced SNP sets $D_{1},\cdots ,D_{K}$, each of which retains a specific high proportion (i.e., 1 − *ϵ*_1_, ⋯, 1 − *ϵ*_*K*_, respectively) of base signals. Step 2: For each reduced SNP set $D_{k}$, train the joint prediction model using the target training sample. The joint model that yields the largest *R*^2^ is selected as the final model and the corresponding SNP effect estimates are denoted as ${\hat{\beta }}^{*}$. Step 3: Apply the estimated effects ${\hat{\beta }}^{*}$ from the final model of Step 2 to the target testing sample to calculate individual PRS.

Fig 1 shows a flow chart of the transfer-learning procedure with FNC marginal screening and joint model training. In Step 1, we apply FNC marginal screening to the base data to extract useful knowledge as follows. First, we pre-fix a sequence of control levels, *ϵ*_1_, …, *ϵ*_*K*_, on FNP, which is the number of false negatives divided by the total number of signals $|S^{+}|$. In this paper, we set *ϵ*_*k*_’s in grid values of {0.02, 0.04, …, 0.4}. Second, for a given *ϵ*_*k*_, we apply the FNC screening procedure of [18] on *p*-values of the base summary statistics and obtain a reduced SNP set $D_{k}$, which is expected to contain a (1 − *ϵ*_*k*_) proportion of base signals in $S^{+}$ as well as some noise SNPs. However, because FNC screening excludes noise SNPs that are distinguishable from the (1 − *ϵ*_*k*_) proportion of signals in $S^{+}$, each $D_{k}$ can be much smaller than the full set of SNPs. More detailed descriptions of FNC screening is provided in the next subsection.

In Step 2, we equally and randomly divide the individual-level target sample into training and testing samples, and use the training sample and Lasso regression to build the PRS model. Specifically, given a reduced SNP set $D_{k}$, *k* = 1, …, *K*, we fit a joint prediction model using regularized regressions, select the regularization parameter using cross-validation or information criterion, and obtain the regularized estimates of SNP effects and compute the model *R*^2^ value. Among the *K* PRS models, the model yields the largest *R*^2^ value is selected as the final model, and the corresponding effect size estimates (denoted by ${\hat{\beta }}^{*}$) are used to construct PRS. In Step 2, working with the FNC reduced SNP set $D_{k}$ instead of the entire SNP set enhances prediction accuracy and computational efficiency of the joint-model training. In Step 3, we apply the estimated effects from the final model ${\hat{\beta }}^{*}$ to the target testing sample and calculate PRS for each testing individual.

The proposed transfer learning procedure is general and can accommodate a spectrum of prediction models such as linear mixed effects models (LMM) [19] and penalized linear regression [20, 21] in Step 2. In this paper, we adopt the popular Lasso regression to obtain sparse effect estimates and utilize cross-validation to determine the hyper-parameters. We denote the whole procedure as FNC+Lasso and show the complete algorithm of FNC+Lasso in Algorithm 1 of S1A Appendix. The computer code for FNC+Lasso, together with complete documentation is publicly available at Github: https://github.com/JessieJeng/FNC-Lasso.

We note that the default implementation of FNC+Lasso at Github does not use LD information. When LD is not accounted for, the total number of base signal variants, denoted as |*S*^+^|, could be overestimated, but the computational time required for estimating |*S*^+^| can be substantially reduced. An overestimated |*S*^+^| would result in more conservative screening results, i.e., more variants would be selected to enter the joint-modeling step. Given the computational gain and the screening results, we opt not to incorporate LD information among SNPs in the screening step.

### FNC marginal screening

The original FNC screening procedure was designed to preserve a high proportion of signals via effectively controlling false negatives [18]. In comparison to other existing false negative control methods [22–24], FNC screening exhibits the capacity to accommodate arbitrary covariance dependencies [18]. FNC screening can be applied as follows. Given a sequence of *p*-values for all the SNPs in the base data: *p*_1_, …, *p*_*m*_, rank the SNPs by their *p*-values such at *p*_(1)_ ≤ *p*_(2)_ ≤ … ≤ *p*_(*m*)_. For each *j* ∈ {1, …, *m*}, calculate

$$\begin{array}{c} {\hat{FNP}}_{j}=\text{max}\left\{1-j/\hat{s}+\left(m-\hat{s}\right)p_{\left(j\right)}\right)/\hat{s},0\}, \end{array}$$ (1)

where $\hat{s}$ is an estimate for $|S^{+}|$, the number signal variants in the base data. Now, for a control level *ϵ*_*k*_, derive

$$\begin{array}{c} j_{k}^{*}=\text{min}\left\{j:{\hat{FNP}}_{j}<\epsilon_{k}\right\}. \end{array}$$ (2)

Then, construct the SNP set $D_{k}$ as the collection of SNPs whose *p*-values are less than or equal to $p_{\left(j_{k}^{*}\right)}$. Repeat the above for each of *ϵ*_1_, …, *ϵ*_*K*_, and obtain the reduced SNP sets *D*_1_, …, *D*_*K*_.

The FNC screening procedure is based on direct estimation for the true FNP if only the top *j* ranked SNPs are selected. Rationale of the estimator in (1) is as follows. When the top *j* variants are selected, the total number of signal variants equals to the sum of true positives (*TP*_*j*_) and false negatives (*FN*_*j*_), i.e. $|S^{+}|=s=TP_{j}+FN_{j}$. Then we have *FNP*_*j*_ = *FN*_*j*_/*s* = 1 − *TP*_*j*_/*s*, and we would need to estimate *s* and *TP*_*j*_ for each *j*.

FNC screening uses $\hat{s}$ from [25] as recommended in [18] to estimate *s*, whose consistency has been proved under arbitrary covariance dependence. For *TP*_*j*_, note that selecting the top *j* variants incurs true positives and false positives with *j* = *TP*_*j*_ + *FP*_*j*_. Then *TP*_*j*_ = *j* − *FP*_*j*_. It is reasonable to use $E\left(FP_{j}\right)=\left(m-\hat{s}\right)p_{\left(j\right)}$ to approximate *FP*_*j*_ based on the null distribution of the p-values. Then *TP*_*j*_ can be approximated by $j-\left(m-\hat{s}\right)p_{\left(j\right)}$ and, consequently, *FNP*_*j*_ can be approximated by $1-j/\hat{s}+\left(m-\hat{s}\right)p_{\left(j\right)}/\hat{s}$ as in Eq (1).

Moreover, because the true FNP_*j*_ is non-increasing with respect to *j*, stopping at the first time ${\hat{FNP}}_{j}$ is less than *ϵ*_*k*_ can result in the smallest subset of variants with FNP controlled at *ϵ*_*k*_ with high probability. This step, as stated in Eq (2), can effectively exclude noise variants that are distinguishable from the (1 − *ϵ*_*k*_) signals. More detailed justifications in theory and simulation of FNC screening can be found in [18].

We note that one can consider all possible p-value thresholds to search for the optimal reduced SNP subset $D_{k^{*}}$ for joint-model training. However, considering all possible thresholds will result in an extremely large number of candidate models, *K*, to be trained in the joint modeling step using the target data. Thus the practical and conceptual benefits of FNC screening are to provide a moderate *K* (e.g., *K* = 20 in our default setting) based on the principle of FN control and to obtain the *K* candidate reduced SNP sets, each of which retains a high proportion of signal variants while minimizes the false positives by selecting the smallest subset of promising variants with respect to a specific FN proportion *ϵ*_*k*_.

## Results

### Simulation design

For the target data in simulation, we obtain the genotype data of Chromosome 21 from the CoLaus/PsyCoLaus study [13, 14]. After data pre-process described in S1C Appendix, the genotype data for Chromosome 21 has *m* = 5053 SNPs. We randomly select *n* individuals to form the target sample, resulting in a genotype matrix *X* consisting of *m* columns and *n* rows. Let *β* be a *m*-dimensional vector of the allelic effects. We assume that *β* is a sparse vector with *β*_*j*_ ≠ 0 for j∈S, where S is the set of causal variants for the target sample. The trait vector *Y* are simulated from *Y* = *Xβ* + *W*, where *W* ∼ *N*_*n*_(0, *I*), *I* is an identity matrix. The simulated target sample is randomly split into training set and testing set with a 1 : 1 ratio. The training set is used to build PRS models using different methods. The testing set is used to evaluate the predictive performances of these methods.

In our main simulations, we assume that $S\subseteq S^{+}$. The base summary data are generated by *Z* ∼ *N*_*m*_(*μ*^+^, Σ/*n*_0_), where *μ*^+^ is the mean vector of *Z*, *n*_0_ represents the base sample size, *Σ* is the correlation matrix of all the *m* = 5053 SNPs, which is estimated from the CoLaus/PsyCoLaus samples. Such marginal effect model has been used in GWAS analysis in e.g. [26]. The mean vector *μ*^+^ has $\mu_{j}^{+}\neq 0$ for $j\in S^{+}$ and 0 otherwise, where $S^{+}$ is the set of base signal variants. In the simulation studies, we consider 3 sets of (*n*_0_, *n*), which are (4000, 2000), (4000, 1000) and (2000, 1000). We control the overlap between base signal variants ($S^{+}$) and target causal variants (S) using a proportion parameter $\delta =\left|S|/\right|S^{+}|$, where |⋅| represents the cardinality of a set. It is expected that the more diverse the base samples are, the smaller the *δ* would be. In the simulation examples, we randomly select 50 SNPs as signal variants in the base sample (i.e., $|S^{+}|=50$) and set the number of target causal variants as |*S*| = 50 × *δ*. We consider *δ* = 0.3, 0.5 and 0.7, representing low, median, and high overlaps between S and $S^{+}$, respectively. The non-zero effect sizes of $\mu_{j}^{+}$ and *β*_*j*_ are generated independently from Uniform(0.05, 0.15) and separately for base and target data. The range of the uniform distribution is calibrated so that *R*^2^ roughly range from 5% to 30% for different methods across different scenarios. The heritability *h*^2^, quantified by *V*(*Xβ*)/*V*(*Y*), is about 0.14, 0.21 and 0.28 for *δ* = 0.3, 0.5 and 0.7, respectively.

We also conduct additional simulations where the nested assumption is not satisfied (i.e., $S⊈S^{+}$) and the effect sizes of the causal/signal variants are generated from more conventional settings, e.g., allowing the true effect sizes of base and target causal variants (denoted by *β*^+^ and *β*, respectively) in the joint-SNP models to be correlated and to be positive or negative, as well as incorporating SNP LD in both mean and variance of the base summary statistics *Z* when generating *Z*. Specifically, we control the overlap proportion between the target causal variants (S) and the base causal variants (denoted by $S_{\beta^{+}}^{+}$) using parameter $\delta^{*}=|S\cap S_{\beta^{+}}^{+}|/|S|.$ We consider $\left|S|=\right|S_{\beta^{+}}^{+}|=50$, δ* = 0.3, 0.5, and 0.7, (*n*_0_, *n*) = (4000, 1000), and *h*^2^ ≈ 0.33. We provide the detailed designs of the additional simulations in S1B Appendix and summarize the data generation schemes in Table 1 for the simulation study.

**Table 1:** Summary of data generation schemes in the simulations. *β*^+^ and *β* are the true effect size of base and target data, respectively, from the joint models; *μ*^+^ is the mean of the summary statistics, typically obtained from marginal models; Σ is the correlation matrix of the *m* = 5, 053 SNPs; *n*_0_ and *n* are the sample size of base and target data, respectively.

	Main Simulations $S\subseteq S^{+}$	Additional Simulations $S⊈S^{+}$
$\begin{array}{c} \text{Causal}\;\text{Variant}\;\text{Effect}\;(\beta_{j}^{+}\text{;}\beta_{j})\\ \text{or}\;\text{Signal}\;\text{Variant}\;\text{Effect}\;(\mu_{j}^{+}) \end{array}$	$\begin{array}{c} \mu_{j}^{+}∼Unif\left(0.05,0.15\right)\\ \beta_{j}\;∼Unif\left(0.05,0.15\right) \end{array}$	$\begin{array}{c} \left[\begin{array}{c} \beta_{j}^{+}\\ \beta_{j} \end{array}]∼N_{2}([\begin{array}{c} 0\\ 0 \end{array}],\tau^{2}[\begin{array}{cc} 1 & \rho \\ \rho & 1 \end{array}]\right)\\ \rho \neq 0\;\text{if}\;j\;\text{shared}\;\text{between}\;\text{base}\;\text{and}\;\text{target;}\\ \rho =0\;\text{otherwise} \end{array}$
Base Summary Data *Z*	*Z* ∼ *N*_*m*_(*μ*^+^, Σ/*n*_0_)	*Z* ∼ *N*_*m*_(*μ*^+^ = Σ*β*^+^, Σ/*n*_0_)
Target Data *Y*	*Y* ∼ *N*_*n*_(*Xβ*, 1)	*Y* ∼ *N*_*n*_(*Xβ*, 1)
Results	Fig 2 and Table 2	S1 Fig and S1 Table

### Performance assessment and baseline methods

We simulate 100 replicates under each simulation scenario, and evaluate the predictive performance, model fitting and model parsimony for the final PRS model. Specifically, we examine two metrics: (1) *R*^2^, which quantifies the outcome variation explained by the PRS on the target testing set; and (2) Akaike Information Criterion (AIC), which evaluates how well the PRS model fits the outcome data and has been used to evaluate polygenic prediction in [27] and [28]. AIC is calculated by *AIC* = 2*q* + *n*_*test*_ ⋅ log(*RSS*/*n*_*test*_)), where *q* is the number of SNPs retained in the PRS model, *n*_*test*_ is the sample size of the target testing set, and *RSS* is the residual sum of square (RSS) calculated on the target testing set. As an ancillary metric, we also report the number of SNPs included in the final PRS model.

We benchmark the proposed FNC+Lasso transfer learning method against five representative methods for PRS analysis, which are Clumping+Thresholding (CT; [5]), lassosum [8], LDpred [9, 11], Lasso [29], and SIS+Lasso. The first three methods use the LD information obtained from Chromosome 21 of the CoLaus/PsyCoLaus data, estimate the effect sizes using base summary data, and are implemented using R package bingsnpr [30] in our numerical studies. In contrast, Lasso only uses the individual-level target data, see for example [21]. The last method, SIS+Lasso, can be viewed as a naive transfer learning procedure that also includes dimension reduction and joint-model training. Instead of using FNC screening and searching adaptively for the optimal reduced SNP set, SIS+Lasso applies sure independence screening (SIS) [31] to the base summary statistics to select the top *n*_*train*_ − 1 SNPs, with *n*_*train*_ being the target training sample size, and then performs Lasso regression on the selected SNPs with the target training data. It is well-known that SIS can be applied to marginal summary statistics to quickly reduce data dimension. We include SIS+Lasso in the baseline methods to evaluate potential advantages of using FNC screening in the transfer learning framework.

### Simulation results

**Fig 2:**
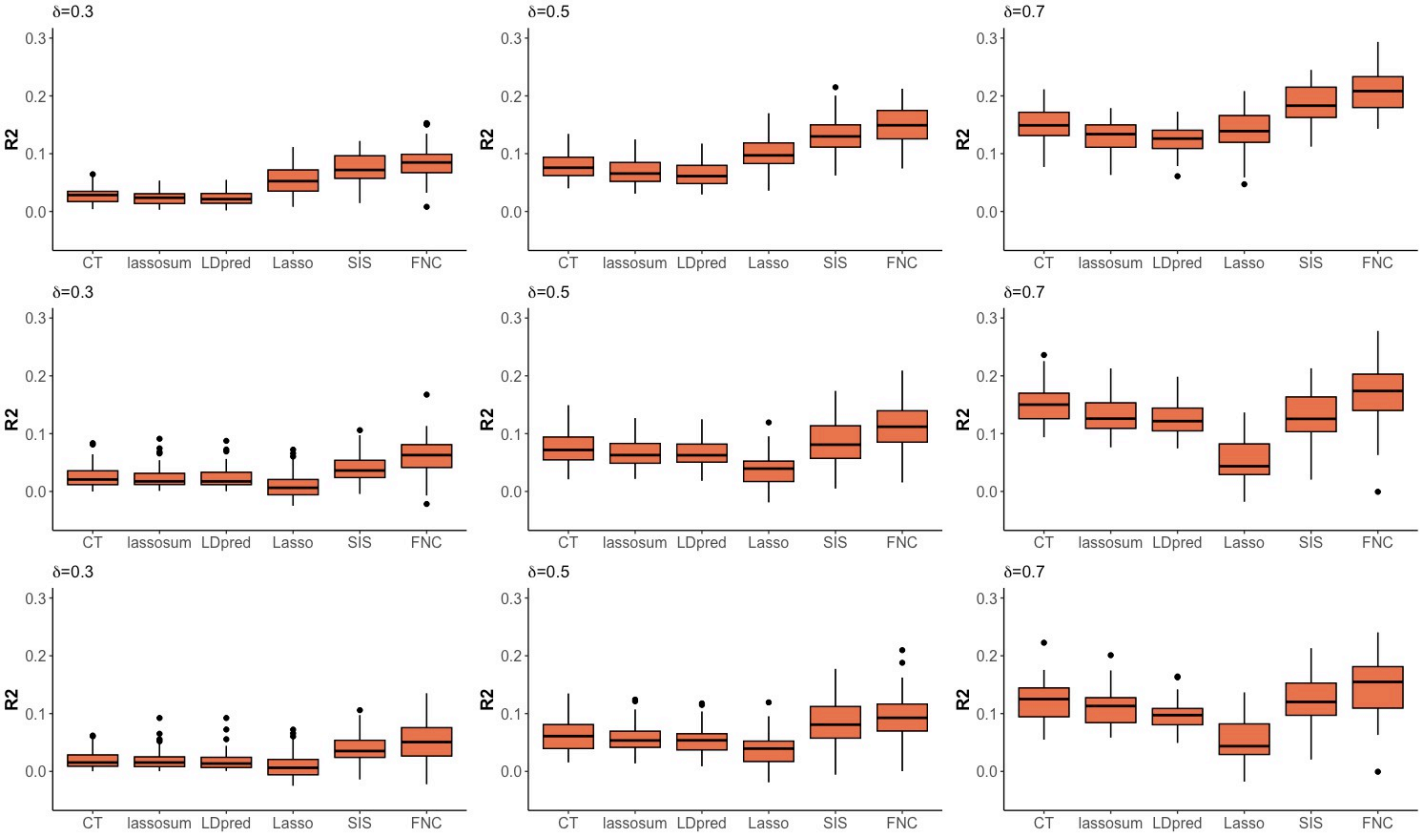
Results of *R*^2^ for prediction accuracy of different PRS methods in the main simulations assuming $S\subseteq S^{+}$. The results are based on 100 simulation replicates under different sample-size combinations of base data (*n*_0_) and target data (*n*), and under different overlap proportion between base signal variants ($S^{+}$) and target causal variants (S), i.e., $\delta \equiv \left|S|/\right|S^{+}|$ with $|S^{+}|=50$. From the top to bottom rows are for (*n*_0_, *n*) = (4000, 2000), (4000, 1000) and (2000, 1000), respectively. In each replication, the target sample is randomly and equally divided into training and testing sets. From the left to right columns are for *δ* = 0.3, 0.5, and 0.7, respectively. Methods considered include Clumping+Thresholding (CT), lassosum, LDpred, Lasso, SIS+Lasso (SIS), and FNC+Lasso (FNC).

Fig 2 shows the boxplots of *R*^2^ from 100 replications under the scenario of $S\subseteq S^{+}$, for (*n*_0_, *n*) = (4000, 2000) (top row), (4000, 1000) (middle row) and (2000, 1000) (bottom row). In each replication, the target sample is randomly and equally divided into training and testing sets. In all scenarios, we observe that FNC+Lasso tend to give the highest *R*^2^ among all methods. We also observe that methods using a trans-learning framework (i.e., FNC+Lasso and SIS+Lasso) generally outperform other baseline methods. The only exception is when the target training sample size is relatively small (e.g., *n* = 1000 and hence *n*_*training*_ = 500) and the overlap between base signals and target causal variants is high (e.g., *δ* = 0.7), where CT, lassosum and LDpred can perform comparable to or better than SIS+Lasso. Nevertheless, FNC+Lasso, which aims to reserve an optimal high proportion of signals during screening, consistently yield better *R*^2^ than SIS+Lasso and other baseline methods.

The relative performances of the non-trans-learning baseline methods are sensitive to the target sample size *n* and the overlap proportion *δ*. Specifically, when target sample size is relatively large (e.g., *n* = 2000; top row of Fig 2), Lasso tends to have higher *R*^2^ than those methods relying on the effect sizes estimated from base data, i.e., CT, lassosum, and LDpred. The gap is more obvious when the overlap proportion *δ* is small. The results suggest that re-estimating SNP effects using target training data (i.e., Lasso) can improve predictive performance when the target sample size is sufficiently large, and that the gain is more substantial when the base signals and target causal variants are less homogeneous (i.e., smaller *δ*).

In contrast, when target sample size is relatively small (e.g., *n* = 1000; middle and bottom rows of Fig 2), CT, lassosum, and LDpred generally outperform Lasso. The gap is more obvious when the overlap proportion *δ* becomes larger. The results suggest that when target sample size *n* (and hence the target training sample size *n*_*train*_) is not sufficiently large, it becomes inefficient to re-estimate SNP effects using target training data, even when the base signals and target causal variants become heterogeneous (e.g., overlap proportion *δ* = 0.3 or 0.5). When the overlap between the base signal set and the target causal set increases, relying on the base effect estimates, which are obtained on a larger sample size *n*_0_, can enhance predictive *R*^2^ (e.g., in the middle and bottom rows, the gap between Lasso and CT/lassosum/LDpred becomes larger as *δ* increases from 0.3 to 0.7). We also observe that the results of (*n*_0_, *n*) = (4000, 1000) and (2000, 1000) are very similar, implying that the base sample size *n*_0_ has less impact on relative performance of the baseline methods.

Table 2 shows AIC and the number of SNPs included in the final PRS model under the scenario of $S\subseteq S^{+}$. A smaller AIC value indicates a better model fitting in simplicity and accuracy. When the overlap proportion is not high (e.g., *δ* = 0.3 and 0.5), FNC+Lasso tends to have the lowest AIC and select the least number of SNPs, closely followed by SIS+Lasso. When *δ* is high (e.g., 0.7), CT tends to have the lowest AIC and select the least number of SNPs, closely followed by FNC+Lasso.

**Table 2:** Results of Akaike Information Criterion (AIC) and number of SNPs in the final PRS model of different methods for assessing model fit and parsimony under the main simulations assuming $S\subseteq S^{+}$.

Overlap (*δ*)	Method	(*n*_0_, *n*) = (4000, 2000)		(*n*_0_, *n*) = (4000, 1000)		(*n*_0_, *n*) = (2000, 1000)	
		Selected SNPs	AIC	Selected SNPs	AIC	Selected SNPs	AIC
0.3	CT	62 (50)	239 (102)	77 (130)	214 (267)	53 (41)	168 (96)
	lassosum	222 (182)	564 (361)	248 (287)	558 (567)	232 (258)	529 (513)
	LDpred	2732 (207)	5583 (419)	2634 (233)	5329 (463)	2454 (283)	4972 (570)
	Lasso	62 (32)	212 (87)	37 (35)	143 (83)	37 (35)	143 (83)
	SIS+Lasso	55 (23)	178 (77)	33 (17)	119 (50)	33 (18)	**119 (51)**
	FNC+Lasso	40 (20)	**131 (65)**	37 (28)	**117 (77)**	35 (29)	**119 (79)**
0.5	CT	54 (13)	274 (63)	59 (54)	198 (114)	51 (21)	188 (58)
	lassosum	187 (97)	551 (205)	173 (114)	430 (234)	151 (91)	391 (188)
	LDpred	2701 (179)	5582 (363)	2753 (219)	5592 (441)	2548 (276)	5187 (554)
	Lasso	107 (38)	356 (101)	57 (41)	215 (85)	57 (41)	215 (85)
	SIS+Lasso	83 (24)	273 (62)	55 (27)	186 (60)	54 (26)	185 (56)
	FNC+Lasso	62 (39)	**214 (109)**	47 (23)	**155 (64)**	43 (25)	**155 (63)**
0.7	CT	51 (9)	271 (57)	50 (7)	**187 (39)**	44 (12)	**193 (42)**
	lassosum	174 (73)	539 (155)	146 (71)	392 (151)	140 (63)	392 (136)
	LDpred	2719 (163)	5637 (339)	2734 (200)	5572 (399)	2576 (279)	5270 (552)
	Lasso	148 (47)	479 (108)	77 (50)	294 (100)	77 (50)	294 (100)
	SIS+Lasso	115 (27)	357 (64)	69 (24)	240 (55)	67 (23)	238 (55)
	FNC+Lasso	79 (54)	**257 (139)**	61 (35)	199 (95)	59 (38)	208 (93)

The reported values are the mean (and standard deviation) based on 100 simulation replicates under different sample-size combinations of base data (*n*_0_) and target data (*n*), and under different overlap proportion between base signal variants ($S^{+}$) and target causal variants (S), i.e., $\delta \equiv \left|S|/\right|S^{+}|$ with $|S^{+}|=50$. Methods considered include Clumping+Thresholding (CT), lassosum, LDpred, Lasso, SIS+Lasso (SIS+Lasso), and FNC+Lasso (FNC+Lasso). Models with the smallest AIC as shown in bold.

The results of the additional simulations considering the non-nested scenario (i.e., $S\subseteq S^{+}$) are summarized in S1 Fig and S1 Table. For *R*^2^ (S1 Fig), we see that across different δ*’s (i.e., overlap proportions between the base and target causal variants) and different *ρ*’s (i.e., effect correlations between the overlapping base and target causal variants), the methods utilizing a trans-learning framework (i.e., FNC+Lasso and SIS+Lasso) generally perform better than or comparable to CT, lassosum, and LDpred; however, the trans-learning methods tend to perform worse than Lasso, especially when there is limited overlap between the base signals and target causal variants. These outcomes are not surprising because leveraging information from the base data cannot enhance the prediction accuracy for the target individuals when a fair proportion of the causal variants are unique to the target population. Between the two transfer-learning methods, FNC+Lasso and SIS+Lasso have similar *R*^2^ across all scenarios, which is perhaps because SIS always selects a fixed large number of SNPs into the joint-model training regardless of the informativeness of base data and consequently, retains some robustness against the base uninformativeness. As the overlap proportion δ* increases from 0.3 to 0.7, the performance of Lasso remains stable, whereas all the other methods exhibit improvement with higher *R*^2^ values; when δ* = 0.7 (i.e., the third column of S1 Fig), FNC+Lasso and SIS+Lasso yield *R*^2^ comparable to Lasso. Finally, the effect correlation *ρ* does not seem to have much impact on the relative performance between FNC+Lasso and the baseline methods, except that when the effect sizes of the base and target causal variants are highly correlated (i.e., *ρ* = 0.9) and the overlap proportion is high (δ* = 0.7), all methods have similar *R*^2^. For model fit and parsimony (S1 Table), SIS+Lasso yields the most parsimonious results with the smallest AIC measures in all settings, closely followed by FNC+Lasso and then Lasso..

### Real data applications

We applied the proposed FNC+Lasso method and the baseline methods to construct the PRSs for plasma lipids, including total cholesterol (CHOL), triglycerides (TRIG), high-density lipoprotein (HDL) and low-density lipoprotein (LDL). Plasma lipids are risk factors for cardiovascular diseases, a leading cause of death worldwide [32], and CHOL, TRIG, HDL and LDL are effective biomarkers and risk factors for heart attack and heart diseases. At present, over 1,900 common SNPs have been shown to be associated with CHOL with different significant levels and the corresponding number for TRIG is 4,002 [33]. However, majority of these variants confer small risk individually and have limited predictive accuracy for lipid traits [34], although the variance proportions explained by PRS for Whites and Hispanics tend to be higher than other populations [35].

Our target data is obtained from the CoLaus/PsyCoLaus GWAS study of the Cohort Lausanne, Switzerland [13, 14], consisting of 5,247 Switzerland Caucasians individuals. We acquired the base data from [36] and [37], which includes samples of general European descents from US, central Europe (i.e., Australia, Denmark, France Germany, Netherlands, Switzerland, UK), southern Europe (Italy) and northern Europe (i.e., Iceland, Finland, and Sweden). The base sample has broader ancestral background and larger sample sizes (i.e., 94,595, 100,184, 99,900 and 95,454 for CHOL, TRIG, LDL and HDL, respectively). We pre-process the genotype data by conducting quality control (QC) and imputation as detailed in S1C Appendix and matched SNPs between the base and target data. The number of matched SNPs for CHOL, TRIG, LDL and HDL are 1,833,233, 1,756,492, 1,833,283 and 1,833,233, respectively.

To construct PRSs, we randomly split the target sample into training and testing sets, each containing half of the target sample. We apply the six methods, i.e., the 5 baseline methods (CT, lassosum, LDpred, Lasso and SIS+Lasso) and the proposed FNC+Lasso. For CT, lassosum and LDpred, the LD information is obtained from the CoLaus/PsyCoLaus samples, and the effect sizes in the PRS model are estimated using the base summary data. In all real data analyses, we include the top 10 principal components for population substructure, sex and age as covariates. We optimize the tuning parameters and obtain the effect size estimates for the final PRS model in training set, and calculate the prediction *R*^2^ of the final PRS model in testing set. To account for the variations involved in the random data splits, we repeat the process using 50 different splitting of training and testing sets. In this part, we use the R package *bigsnpr* for Lasso to ensure computational efficiency with whole genome SNPs.

**Fig 3:**
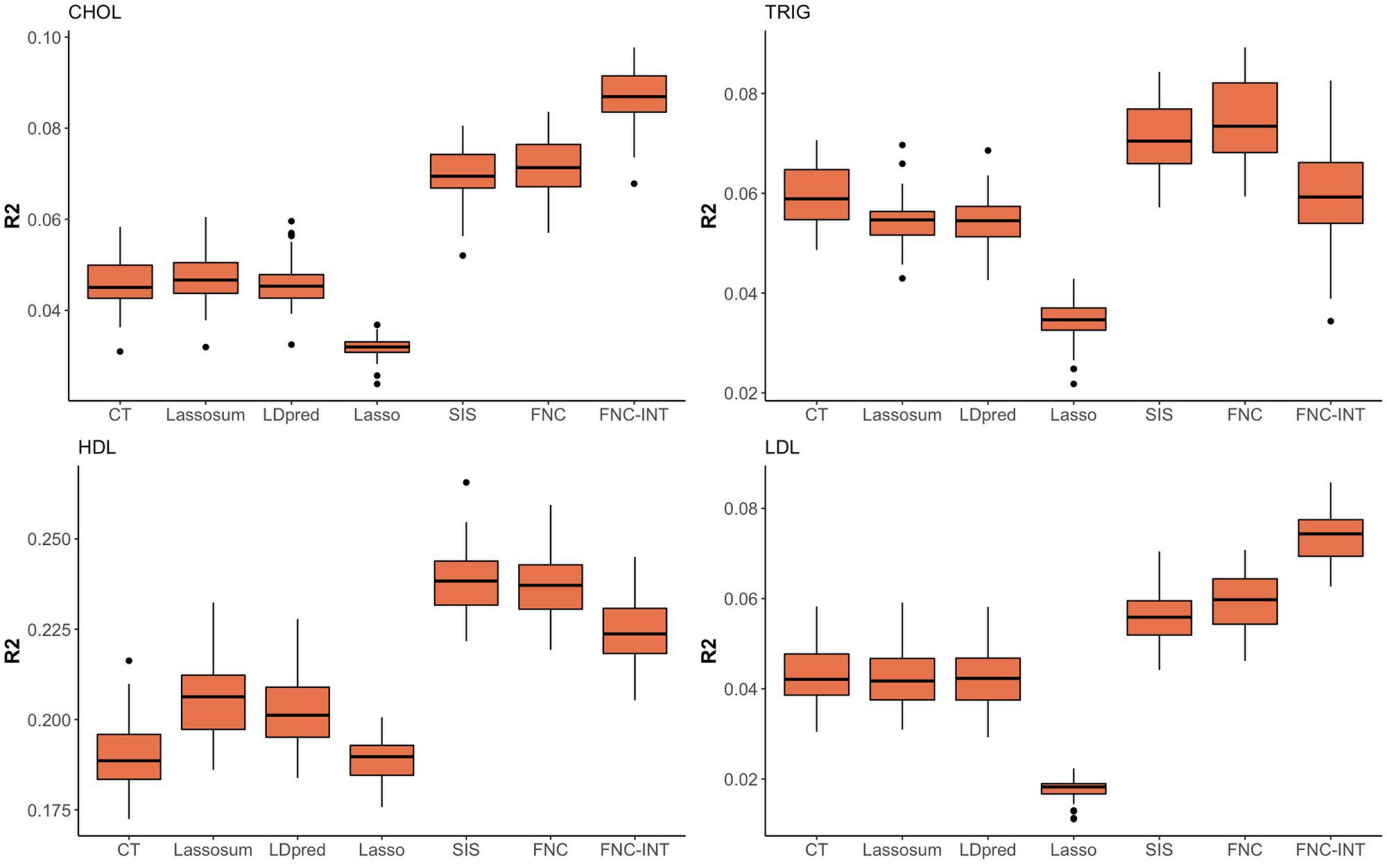
*R*^2^ values for prediction accuracy of different PRS methods. Methods considered include Clumping+Thresholding (CT), lassosum, LDpred, Lasso, SIS+Lasso (SIS), FNC+Lasso (FNC), and interaction model based on FNC+Lasso identified SNPs (FNC-INT) for four lipid traits.

Table 3 shows the *R*^2^, AIC and number of selected SNPs of different methods. Across all four traits, the proposed FNC+Lasso yields more robust performance in *R*^2^ and AIC by yielding the best or comparable values to the best-performing methods, while the best-performing methods vary depending on traits, *R*^2^ and AIC. For example, for CHOL and TRIG, FNC+Lasso has the highest *R*^2^ and lowest AIC. For HDL and LDL, FNC+Lasso and SIS+Lasso have the highest *R*^2^ while lassosum has the smallest AIC. We also observe that although giving reasonable AICs, the classical Lasso has the lowest *R*^2^ for all four traits, suggesting that re-training a PRS model using Lasso on all SNPs with target training sample does not yield ideal predictive accuracy. Incorporating a screening procedure using base data (i.e., trans-learning-based SIS+Lasso and FNC+Lasso) can substantially enhance the predictive *R*^2^ of Lasso. Similar to what is observed in the main simulations, Fig 3 shows that the trans-learning based methods consistently yield higher *R*^2^ than other baseline methods for all four traits, and FNC+Lasso tends to have higher *R*^2^ than SIS+Lasso. We also observe that FNC+Lasso tends to use the less number of SNPs while achieving a higher predictive *R*^2^.

**Table 3:** Prediction *R*^2^, number of selected SNPs, AIC, and computing time of different PRS methods for the CoLaus/PsyCoLaus analysis of four lipid traits.

Trait	Method	Predictive *R*^2^	Selected SNPs	AIC	Computing Time (sec)
CHOL	CT	4.6% (0.6%)	50729 (122314)	101572 (244630)	1143 (101)
	lassosum	4.7% (0.5%)	957 (6514)	2032 (13023)	7320 (1)
	LDPred	4.6% (0.5%)	928258 (17319)	1856637 (34651)	7200 (1)
	Lasso	3.2% (0.2%)	315 (276)	782 (54)	326 (69)
	SIS + Lasso	7% (0.6%)	344 (47)	735 (88)	**15 (1)**
	FNC + Lasso	**7.2% (0.7%)**	229 (28)	**499 (79)**	616 (36)
TRIG	CT	5.9% (0.6%)	118645 (194875)	238203 (389599)	763 (272)
	lassosum	5.4% (0.5%)	832732 (54187)	1666391 (108373)	6900 (1)
	LDPred	5.4% (0.5%)	1183748 (59460)	2368424 (118933)	6781 (1)
	Lasso	3.4% (0.4%)	260 (257)	1503 (694)	143 (21)
	SIS + Lasso	7% (0.7%)	239 (44)	1359 (413)	**13 (1)**
	FNC + Lasso	**7.4% (0.8%)**	170 (21)	**1211 (402)**	533 (39)
HDL	CT	19% (1%)	15 (16)	-4865 (70)	1826 (310)
	lassosum	20.6% (1%)	9 (0)	**-4929 (62)**	7200 (1)
	LDPred	20.3% (1%)	865651 (3866)	1726365 (7729)	7800 (1)
	Lasso	18.9% (0.6%)	699 (361)	-3494 (732)	110 (27)
	SIS + Lasso	**23.8% (0.9%)**	365 (51)	-4326 (106)	**18 (5)**
	FNC + Lasso	**23.7% (0.9%)**	205 (38)	-4642 (96)	596 (76)
LDL	CT	4.2% (0.6%)	125 (302)	-258 (624)	961 (39)
	lassosum	4.2% (0.6%)	109 (0)	**-289 (64)**	8280 (1)
	LDPred	4.2% (0.6%)	893486 (27412)	1786465 (54829)	8881 (1)
	Lasso	1.8% (0.2%)	356 (290)	270 (587)	508 (151)
	SIS + Lasso	5.6% (0.5%)	331 (49)	117 (122)	**17 (3)**
	FNC + Lasso	**5.9% (0.6%)**	171 (26)	-211 (88)	579 (59)

The reported values are the mean (and standard deviation) from 50 random splits for each of the four lipid traits, including total cholesterol (CHOL), triglycerides (TRIG), high-density lipoprotein (HDL) and low-density lipoprotein (LDL). Methods considered include Clumping+Thresholding (CT), lassosum, LDpred, Lasso, SIS+Lasso (SIS+Lasso), and FNC+Lasso (FNC+Lasso). Best performing results across different methods are shown in bold.

Table 3 also shows that the proposed FNC+Lasso is computationally efficient. It is slower than SIS+Lasso and Lasso because FNC+Lasso involves a grid search to determine two hyper-parameters (FN proportion *ϵ*_*k*_ and the shrinkage parameter of Lasso). However, the required computing time is still less than CT, lassosum and LDpred, e.g., FNC+Lasso can be 1.4∼15 times faster than these baseline methods.

The parsimonious modeling of FNC+Lasso could foster interpretability and facilitate more complex modeling in downstream analyses, such as incorporating SNP-SNP interactions in polygenic prediction models. Here we explore pairwise interactions of the SNPs selected in FNC+Lasso on the lipid traits. To avoid potential overestimation of the interaction effects, we fit an linear regression model that satisfies strong hierarchy (i.e., whenever a SNP-SNP interaction is estimated to be non-zero by Lasso, the main effects of both SNPs are also included in the model) so that the interaction effects are not identified due to their collinearity with the omitted main effects. This procedure utilizes hierarchical group-Lasso regulation introduced in [38] and can be applied using the R package *glinternet*. We investigate the predictive *R*^2^ performance of the interaction PRS model based on FNC-Lasso selected SNPs (denoted as FNC-INT) for the four traits. The results in Fig 3 show that incorporating SNP-SNP interaction terms can further increase the predictive performance for CHOL and LDL, although interactions do not help in TRIG and HDL. Recent studies have gain valuable insights into the functional consequences of some gene-gene interactions on LDL through systematic experiments that simultaneously knockdown candidate gene pairs in cultured HeLa cells [39]. Further follow-up studies could relate our identified SNP-SNP pairs to candidate genes and gene pairs through eQTL analysis and evaluate their roles in biological pathways of lipid traits.

## Conclusion and discussion

Inspired by the idea of transfer learning, the proposed FNC+Lasso procedure aims to leverage the base data information for target data prediction modeling, where the target data may or may not have similar ancestral background as the base data. FNC+Lasso utilizes a unique false negative control strategy to extract useful information from the base data, and applies the extracted information to re-train the PRS models using target data in a statistically and computationally efficient fashion. The benefits are multi-fold: (1) FNC screening leverages the base information to effectively retain a high proportion of base signal variants and yields a moderate number of candidate joint models, which reduces the cost in model re-training. (2) Only the identities of SNPs, not their effect sizes, are carried over to joint modeling, which encourages accurate PRS estimation and prediction for the target samples. (3) Through the efficient exclusion of noise variants by FNC screening, dimensions of the candidate joint models are largely reduced, which helps to alleviate the challenges associated with the “large p small n” joint modeling and leads to enhanced prediction accuracy. (4) The resulting PRS model of FNC+Lasso are parsimonious with better fit, which facilitates model interpretation and complex modeling in follow-up analyses.

The proposed PRS transfer learning framework can accommodate continuous and categorical traits by applying linear or logistic models in the joint modeling step. It is also applicable to target data with only summary statistics available. In that case, the Lasso step on individual-level target data can be replaced by using the lassosum algorithm with GWAS summary statistics from target data. Just like lassosum, the specific summary statistics needed would include: (1) the LD matrix from reference panel with similar ancestral background as the target samples, and (2) the SNP-phenotype correlation from target summary statistics.

FNC+Lasso adopts a transfer learning framework and relies on the nested assumption of $S\subseteq S^{+}$, i.e., the target causal variants S are nested within the base signal variants $S^{+}$. In real practice, such nested assumption could be satisfied by adopting multi-ancestry GWAS base data, e.g., using the large-scale meta-analysis from Global Biobank Meta-analysis Initiative as discussed in Wang et al. [17]. Using simulations, we explore the impact of overlapping proportion $\delta \equiv \left|S|/\right|S^{+}|$ under the scenario of $S\subseteq S^{+}$. We observed that when *δ* is low (i.e., heterogeneous effect patterns between base and target data), re-training PRS models with sufficient target samples can achieve better predictive performance. When *δ* is high (i.e., similar effect patterns between base and target data), utilizing effect estimates from base data can ensure higher predictive accuracy, especially when target samples are of limited size. Our numerical studies also show that FNC+Lasso adapts to overlap level—FNC+Lasso ensures effective PRS joint-model training even with limited target samples, and results in a more robust and better predictive performance than the baseline methods across a range of *δ* values and different base/target sample sizes.

We also conduct additional simulations where the nested assumption is not satisfied. The results show that FNC+Lasso could still outperform existing PRS methods (e.g., C+T, lassosum and LDpred), has similar *R*^2^ as SIS+Lasso, but has smaller *R*^2^ than Lasso. However, Lasso exhibits unstable performance across different nested and non-nested scenarios in our numerical studies. Given that the actual informativeness level of base data on the target causal variants is unknown and can vary from trait to trait in real applications, we would recommend to use methods utilizing a transfer learning framework (i.e., FNC+Lasso and SIS+Lasso) for PRS analysis. If multi-ancestry GWAS base data could be used, FNC+Lasso may provide further improvement upon SIS+Lasso.

The PRS models of FNC+Lasso tend to be more parsimonious compared to baseline models while maintaining a comparable or higher *R*^2^. A parsimonious PRS model that consists of key SNPs and excludes noise SNPs could aid the interpretablity of PRS and help to inform underlying molecular and functional basis, such as by examining the SNPs involved in the PRS model as well as those SNPs in LD with them to identify biologically important variants or to identify hub genes that capture larger polygenicity [40]. By focusing on the SNPs in a parsimonious PRS model, it also allows further exploration of non-additive PRS models, such as the SNP-SNP interaction PRS model considered in the CoLaus/PsyCoLaus data analysis. PRS models incorporating epistasis have been challenging because existing PRS models tend to be comprised of a large number of SNPs, and the corresponding interactions quickly leads to a prohibitive number of predictors in the model. While machine-learning PRS methods such as random forest have been proposed to account for unknown epistasis [41], it remains an open question how the base data information can be incorporated into these learning approaches. Complex models based on FNC+Lasso SNPs may offer an alternative for constructing non-additive PRS.

## Supporting information

S1 Appendix FNC+Lasso algorithm, details of additional simulations, and preprocess of the CoLaus/PsyCoLaus GWAS data. Section A shows the FNC+Lasso algorithm. Section B presents the designs and results of the additional simulations. Section C describes the QC and imputation of the CoLaus/PsyCoLaus GWAS data.
(PDF)

S1 FigResults of *R*^2^ for prediction accuracy of different PRS methods in the additional simulations assuming $S⊈S^{+}$, with (*n*_0_, *n*) = (4000, 1000), $\left|S|=\right|S_{\beta^{+}}^{+}|=50$, δ* = 0.3, 0.5 and 0.7, and *ρ* = 0.5, 0.7 and 0.9.(PDF)

S1 TableResults of Akaike Information Criterion (AIC) and number of SNPs in the final PRS model of different methods for assessing model fit and parsimony in the additional simulations assuming $S⊈S^{+}$, with (*n*_0_, *n*) = (4000, 1000), $\left|S|=\right|S_{\beta^{+}}^{+}|=50$, δ* = 0.3, 0.5 and 0.7, and *ρ* = 0.5, 0.7 and 0.9.(PDF)

S2 Table Numerical data underlying graphs and tables. (XLSX)
