# Supplementary material for: Transfer learning with false negative control improves polygenic risk prediction
Source: PLoS Genet. 2023 Nov 27;19(11):e1010597. doi: 10.1371/journal.pgen.1010597 (PMC10723713; doi:10.1371/journal.pgen.1010597)
Supplement: S1 Appendix — Section A shows the FNC+Lasso algorithm. Section B presents the designs and results of the additional simulations. Section C describes the QC and imputation of the CoLaus/PsyCoLaus GWAS data. (PDF) [file pgen.1010597.s001.pdf]

Transfer Learning with False Negative Control Improves Polygenic Risk Prediction

Xinge Jessie Jeng, Yifei Hu, Vaishnavi Venkat, Tzu-Pin Lu and Jung-Ying Tzeng

S1 Appendix.

A. Algorithm of FNC+Lasso

Algorithm 1: FNC+Lasso

Input: SNP p-values $\{p_1, ..., p_m\}$ of base summary statistics,
phenotype vector $\mathbf{y}$ of target training set with length n_{train} (target training size),
genotype matrix $\mathbf{X}_{n_{train} \times m} = [X_1, \cdots, X_m]$ of target training set,
user-specified false-negative proportion (FNP) control levels $\{\epsilon_1, ..., \epsilon_K\}$, and
estimated number of base signals $\hat{s}$;

Output: estimated effect sizes $\hat{\beta}^* = \{\hat{\beta}_1^*, ..., \hat{\beta}_q^*\}$, where q is the number of SNPs in the reduced SNP set yielding the highest R^2;

```
Rank the p-values obtained from base data so that p_(1) < p_(2) < ... < p_(m);
for k = 1 to K do                          // k indexes for the FNP control level ε_k
    // STEP 1: FNC screening
    for j = 1 to m do                                        // j indexes for SNP
        compute FNP^_(j) = max{1 − j/ŝ + (m − ŝ)p_(j)/ŝ, 0};
        ;                       // FNP^_(j) is the estimated FNP if select top j SNPs
    end
    initialize ℓ = 1;
    while FNP^_(ℓ) ≥ ε_k do
        ℓ = ℓ + 1;
    end
    D_k = {i : p_i < p_(ℓ)} ;           // D_k is the reduced SNP set with FNP set at ε_k
    // STEP 2: Lasso
    given D_k, fit regularized regression y = Σ_{j∈D_k} X_j β_j using Lasso with cross-validation
      on the target training data to obtain the estimated effect β^(k), and compute the
      corresponding R_k^2;
end
k* = arg max_k R_k^2 ;  // k* associated with the SNP set D_{k*} that has max R^2 value
Return: β^* = β^(k*);
```

B. Designs of Additional Simulations with $\mathcal{S} \subseteq \mathcal{S}^+$ not Satisfied

We conduct additional simulations in which the base signal variants $\mathcal{S}^+$ and target causal variants $\mathcal{S}$ are only partially overlapped. We consider $(n_0, n) = (4000, 1000)$, where n_0 and n are the base

sample size for base data and target data, respectively. We use $\mathcal{S}_{\beta+}^{+}$ to denote the set of base causal variants, and control the proportion of the target causal variants ($\mathcal{S}$) overlapping with the base causal variants ($\mathcal{S}_{\beta+}^{+}$) using $\delta^* = \left|\mathcal{S} \cap \mathcal{S}_{\beta+}^{+}\right| / |\mathcal{S}|$. We consider $|\mathcal{S}| = |\mathcal{S}_{\beta+}^{+}| = 50$ and $\delta^* = 0.3$, 0.5, and 0.7.

We randomly select the overlapping and non-overlapping SNPs for $\mathcal{S}_{\beta+}^{+}$ and $\mathcal{S}$ from the $m = 5053$ SNPs. For non-overlapping SNPs, we generate their effect size independently from $N(0, \tau^2)$. When generating the effect size for overlapping SNPs, we adopt a more conventional and realistic setting, where the base and target causal effects are correlated, the effect sizes can be positive or negative, and SNP LD is incorporated in both mean and variance of the base summary statistics Z. That is, for an overlapping SNP j, its effect sizes $(\beta_j^+, \beta_j) \sim N((0,0), \Theta)$, where Θ is a 2×2 matrix with the diagonal terms as τ^2 and the off-diagonal terms as $\rho \cdot \tau^2$. Here β_j^+ and β_j are the true effect size of causal variant j in the joint model for the base data and target data, respectively. We then generate the summary statistics Z of marginal models by $Z \sim N_m(\Sigma\beta^+, \Sigma/n_0)$. Finally we simulate the target trait Y from $N_n(X\beta, 1)$. We consider $\tau = 0.1$ and $\rho = 0.5$, 0.7 and 0.9. The heritability h^2, quantified by $V(X\beta)/V(Y)$, is about 0.33 in all simulations.

C. Quality control (QC) and Imputation of the CoLaus/PsyCoLaus GWAS data

We obtain target data from the CoLaus/PsyCoLaus study. From the raw data, we remove extremely rare SNPs with minor allele frequency (MAF) less than 0.01 and p-value of Hardy-Weinberg Equilibrium (HWE) using Fisher's exact test lower than 10^{-6}. As suggested in [1], we also remove individuals with excessive heterozygosity rates by first computing F coefficient estimates for assessing heterozygosity and then excluding individuals with F coefficients more than three standard deviation units from the mean. Finally, we exclude one individual of each relative pairs whose PLINK IBD proportion estimates are greater than 0.2. The last two steps (i.e., removal of excessive heterozygosity and related samples) are based on a temporally pruned SNPs list with a window size of 200 variants, a step size of 50 variants and a threshold for LD R^2 of 0.25.

We impute the genotypes of the QC'ed CoLaus/PsyCoLaus data using the Michigan Imputation Server (https://imputationserver.sph.umich.edu/). Specifically, we first lift over the QC'ed SNPs from hg18 to hg19 and then impute genotypes using minimac4 with the HRC r1.1 EUR population as the reference panel. We conduct post-imputation QC using the same MAF and HWE procedures as for the raw data as well as removing SNPs with imputation Rsq less than 0.8. These SNPs are then lifted over to hg38 so to be consistent with the Genome Build of the base data; after lifting over, there are 5,768,004 SNPs.

For the phenotypes of CHOL, TRIG, LDL, and HDL, we obtained base datasets from the large-scale GWAS studies of [2] and [3]. From the raw data, we remove SNPs with small MAFs and HWE departure using the same criteria as in the target data. We then match SNPs between the base data and target data by their genomic positions of hg38, retain overlapping SNPs between the two datasets, and flip and reverse some mismatched SNPs.

References

[1] Choi SW, Mak TSH, O'Reilly PF. Tutorial: a guide to performing polygenic risk score analyses. Nature protocols. 2020;15(9):2759–2772.

[2] Teslovich TM, Musunuru K, Smith AV, Edmondson AC, Stylianou IM, Koseki M, et al. Biological, clinical and population relevance of 95 loci for blood lipids. Nature. 2010;466(7307):707–713.

[3] Willer CJ, Schmidt EM, Sengupta S, Peloso GM, Gustafsson S, Kanoni S, et al. Discovery and refinement of loci associated with lipid levels. Nature genetics. 2013;45(11):1274.
