# Supplementary material for: Transfer learning with false negative control improves polygenic risk prediction
Source: PLoS Genet. 2023 Nov 27;19(11):e1010597. doi: 10.1371/journal.pgen.1010597 (PMC10723713; doi:10.1371/journal.pgen.1010597)
Supplement: S1 Fig — (PDF) [file pgen.1010597.s002.pdf]

Transfer Learning with False Negative Control Improves Polygenic Risk Prediction
Jeng et al. (2023)

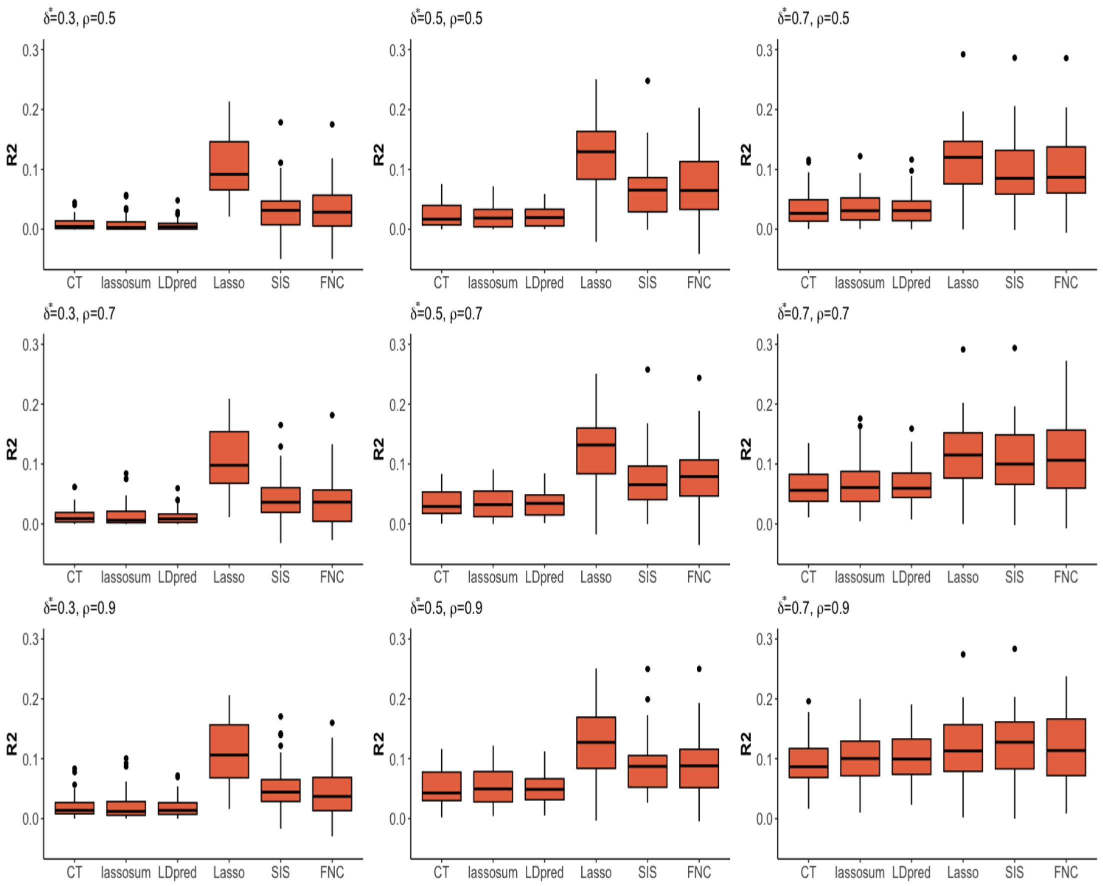

S1 Fig. Results of R^2 for prediction accuracy of different PRS methods in the additional simulations assuming $\mathcal{S} \not\subseteq \mathcal{S}^+$. The results are based on 100 simulation replicates with $(n_0, n) = (4000, 1000)$ and $|S| = |S^+_{\beta+}| = 50$, where $\mathcal{S}$ and $\mathcal{S}^+_{\beta+}$ are the set of target causal variants and the set of base causal variants, respectively. The left to right columns are for different overlap proportions between the base and target causal variants, with $\delta^* \equiv \left|\mathcal{S} \cap \mathcal{S}^+_{\beta+}\right| / |\mathcal{S}| = 0.3$, 0.5, and 0.7, respectively. The top to bottom rows are for different effect correlation between the overlapping base and target causal variants, with $\rho = 0.5$, 0.7 and 0.9, respectively. In each replication, the target sample is randomly and equally divided into training and testing sets. Methods considered include Clumping+Thresholding (CT), lassosum, LDpred, Lasso, SIS+Lasso (SIS), and FNC+Lasso (FNC).
