# Supplementary material for: Transfer learning with false negative control improves polygenic risk prediction
Source: PLoS Genet. 2023 Nov 27;19(11):e1010597. doi: 10.1371/journal.pgen.1010597 (PMC10723713; doi:10.1371/journal.pgen.1010597)
Supplement: S1 Table — (PDF) [file pgen.1010597.s003.pdf]

Overlap δ^*	Method	$\rho = 0.5$ Selected SNPs	$\rho = 0.5$ AIC	$\rho = 0.7$ Selected SNPs	$\rho = 0.7$ AIC	$\rho = 0.9$ Selected SNPs	$\rho = 0.9$ AIC
0.3	CT	224 (519)	628 (1040)	186 (321)	551 (641)	243 (508)	661 (1013)
	lassosum	1030 (1509)	2241 (3025)	1025 (1379)	2227 (2764)	1026 (1278)	2228 (2563)
	LDpred	3335 (1464)	6852 (2933)	3294 (1436)	6767 (2874)	3167 (1452)	6511 (2905)
	Lasso	106 (59)	340 (140)	107 (60)	341 (142)	105 (55)	336 (130)
	SIS+Lasso	33 (21)	**233 (69)**	36 (22)	**236 (71)**	37 (20)	**234 (67)**
	FNC+Lasso	66 (32)	302 (90)	66 (30)	298 (84)	70 (30)	304 (84)
0.5	CT	211 (437)	606 (876)	167 (302)	514 (606)	189 (388)	551 (775)
	lassosum	766 (1131)	1718 (2258)	673 (974)	1526 (1945)	751 (1097)	1674 (2188)
	LDpred	3255 (1315)	6697 (2627)	3058 (1279)	6279 (2554)	2964 (1385)	6101 (2770)
	Lasso	99 (46)	327 (104)	95 (46)	321 (103)	95 (46)	319 (103)
	SIS+Lasso	30 (18)	**222 (53)**	33 (17)	**225 (55)**	37 (17)	**225 (57)**
	FNC+Lasso	58 (26)	273 (64)	60 (23)	275 (63)	64 (26)	279 (69)
0.7	CT	103 (198)	371 (399)	139 (250)	430 (500)	107 (156)	348 (314)
	lassosum	735 (1011)	1636 (2029)	540 (836)	1230 (1675)	538 (902)	1206 (1809)
	LDpred	2980 (1467)	6125 (2940)	2613 (1361)	5377 (2730)	2689 (1487)	5508 (2982)
	Lasso	96 (48)	314 (94)	94 (49)	311 (96)	94 (50)	312 (100)
	SIS+Lasso	45 (21)	**223 (52)**	49 (25)	**226 (56)**	52 (23)	**223 (53)**
	FNC+Lasso	74 (35)	281 (80)	77 (32)	281 (78)	85 (30)	291 (76)

S1 Table. Results of Akaike Information Criterion (AIC) and number of SNPs in the final PRS model of different methods for assessing model fit and parsimony in the additional simulations assuming $\mathcal{S} \not\subseteq \mathcal{S}^+$. The reported values are the mean (and standard deviation) based on 100 simulation replicates with $(n_0, n) = (4000, 1000)$ and $|S| = |S^+_{\beta+}| = 50$, where $\mathcal{S}$ and $\mathcal{S}^+_{\beta+}$ are the set of target causal variants and the set of base causal variants, respectively. Parameter $\delta^* = \left|\mathcal{S} \cap \mathcal{S}^+_{\beta+}\right| / |\mathcal{S}|$ is the overlap proportion between the target causal variants and the base causal variants, and ρ is the effect correlation between the overlapping base and target causal variants. Methods considered include Clumping+Thresholding (CT), lassosum, LDpred, Lasso, SIS+Lasso (SIS+Lasso), and FNC+Lasso (FNC+Lasso). The smallest AIC across different methods under a given simulation scenario are shown in bold.
